# Phylogenetic utility of ribosomal genes for reconstructing the phylogeny of five Chinese satyrine tribes (Lepidoptera, Nymphalidae)

**DOI:** 10.3897/zookeys.488.9171

**Published:** 2015-03-19

**Authors:** Mingsheng Yang, Yalin Zhang

**Affiliations:** 1 Key Laboratory of Plant Protection Resources and Pest Management of Ministry of Education, Entomological Museum, Northwest A&F University, Yangling, Shaanxi 712100, China

**Keywords:** Butterfly, molecular systematics, Bayesian inference, Satyrinae

## Abstract

Satyrinae is one of twelve subfamilies of the butterfly family Nymphalidae, which currently includes nine tribes. However, phylogenetic relationships among them remain largely unresolved, though different researches have been conducted based on both morphological and molecular data. However, ribosomal genes have never been used in tribe level phylogenetic analyses of Satyrinae. In this study we investigate for the first time the phylogenetic relationships among the tribes Elymniini, Amathusiini, Zetherini and Melanitini which are indicated to be a monophyletic group, and the Satyrini, using two ribosomal genes (*28s rDNA* and *16s rDNA*) and four protein-coding genes (*EF-1α*, *COI*, *COII* and *Cytb*). We mainly aim to assess the phylogenetic informativeness of the ribosomal genes as well as clarify the relationships among different tribes. Our results show the two ribosomal genes generally have the same high phylogenetic informativeness compared with *EF-1α*; and we infer the *28s rDNA* would show better informativeness if the *28s rDNA* sequence data for each sampling taxon are obtained in this study. The placement of the monotypic genus *Callarge* Leech in Zetherini is confirmed for the first time based on molecular evidence. In addition, our maximum likelihood (ML) and Bayesian inference (BI) trees consistently show that the involved Satyrinae including the Amathusiini is monophyletic with high support values. Although the relationships among the five tribes are identical among ML and BI analyses and are mostly strongly-supported in BI analysis, those in ML analysis are lowly- or moderately- supported. Therefore, the relationships among the related five tribes recovered herein need further verification based on more sampling taxa.

## Introduction

The butterfly subfamily Satyrinae, comprising approximately 2,500 described extant species, is amongst the most diverse groups in insects (Ackery et al. 1999). Recently, Marín et al. (2011) summarized the findings of systematic studies on this group (Peña et al. 2006; Peña and Wahlberg 2008; Wahlberg et al. 2009), proposing that the Satyrinae could be divided into nine tribes. However, phylogenetic relationships among them remain mostly unresolved despite they are assigned to four groups (Marín et al. 2011): group one consisting of two Neotropical Morphini and Brassolini; group two including Elymniini, Amathusiini, Zetherini, Dirini and Melanitini; group three including only the Neotropical Haeterini; and group four comprising the speciose Satyrini distributed worldwide. Regarding the group two, phylogenetic relationships of its five tribes remain unresolved except for the well-defined sister relationship of Dirini and Melanitini (Peña et al. 2006; Peña and Wahlberg 2008; Wahlberg et al. 2009; Price et al. 2011; see the figure 1 in Marín et al. 2011). The phylogenetic uncertainty among them can be mainly exhibited in two aspects: one is the weakly supported nodes bearing them; and another is the unstable topologies of trees conducted by different analysis methods (Peña et al. 2006; Peña and Wahlberg 2008; Wahlberg et al. 2009).

It is widely accepted that selecting suitable genetic markers is of great importance in study of molecular systematics. In previous phylogenetic studies on the tribe level relationships of Satyrinae, the protein-coding genes (e.g., mitochondrial *COI*, and a number of nuclear genes) have been the main source of phylogenetic information (Peña et al. 2006; Peña and Wahlberg 2008; Wahlberg et al. 2009; Price et al. 2011). However, the ribosomal genes, to date have been never considered. The ribosomal genes have already been proven to be informative for phylogenetic analyses in other butterfly groups (e.g., *16s rDNA* in Kim et al. 2010; *28s rDNA* and *18s rDNA* in Jiang et al. 2013).

In order to test the phylogenetic utility of the ribosome genes for constructing the tribe level relationships of Satyrinae which have not been resolved based on morphological and protein-coding sequence data, two ribosomal genes (*16s rDNA* and *28s rDNA*) as well as four additional protein-coding genes (*COII*, *Cytb*, *COI* and *EF-1α*) are used in our study to reconstruct the phylogeny of the Elymniini, Amathusiini, Zetherini, Melanitini and Satyrini which represent all the major lineages of Chinese satyrines. Besides, we further clarify the taxonomic placement of the *Callarge* Leech, a satyrine genus which has never been included in previous molecular studies.

## Materials and methods

### Taxon sampling

A total of 30 species were included in the analyses (Table 1). Of these, the 21 in-group species represent all the five satyrine tribes occurring in China. In consideration of previous studies (Freitas and Brown 2004; Peña et al. 2006), other nine species of six subfamilies (Libytheinae, Danainae, Apaturinae, Biblidinae, Calinaginae and Charaxinae) of the family Nymphalidae were selected as outgroup taxa. Among them, *Libythea myrrha* Fruhstorfer of Libytheinae was used to root the resulting phylogenetic trees, since Libytheinae is widely accepted as the sister group to the rest Nymphalidae (e.g., Ackery et al. 1999; Freitas and Brown 2004; Peña et al. 2006; Peña and Wahlberg 2008). The butterflies studied stem from the specimens in Entomological Museum of Northwest A&F University (NWAFU), Yangling, China. Details of the sampling are presented in Table 1.

**Table 1.** Samples used for molecular analyses in this study together with relevant information.

Subfamily	Tribe	Species	Specimen voucher	Collecting locality	GenBank accession number					
					*COI*	*COII*	*Cytb*	*16s rDNA*	*EF-1α*	*28s rDNA*
Libytheinae		*Libythea myrrha*	limyr1	China: Yunnan, Jinghong	KC158418*	KJ777775	KJ805831	KJ777730	KJ805856	KJ777756
Danainae	Danaini	*Danaus genutia*	dagen1	China: Yunnan, Hekou	KF226386*	KJ777776	KJ805832	KJ777731	KJ805857	KJ777757
		*Parantica sita*	pasit1	China: Yunnan, Rili	NC_024412*	KJ777777	KJ805833	KJ777732	KJ805858	KJ777758
		*Euploea mulciber*	eumul	China: Yunnan, Lincang	NC_016720*	KJ777778	KJ805834	KJ777733	KJ805859	KJ777759
Apaturinae		*Apatura ilia*	apili1	China: Hunan, Zhangjiajie	NC_016062*	KJ777779	KJ805835	KJ777734	KJ805860	KJ777760
Biblidinae	Biblidini	*Ariadne merione*	armer1	China: Yunnan, Lincang	KC755827*	KJ777780	KJ805836	KJ777735	KJ805861	KJ777761
Calinaginae		*Calinaga davidis*	cadav1	China: Sichuan, Mt. Qingchengshan	NC_015480*	KJ777781	KJ805837	KJ777736	KJ805862	n.a.
Charaxinae	Charaxini	*Charaxes bernardus*	chber1	China: Yunnan, Hekou	EF534101*	KJ777782	KJ805838	KJ777737	KJ805863	n.a.
		*Polyura eudamippus*	poeud1	China: Sichuan, Pinwu	AB855881*	KJ777783	KJ805839	KJ777738	KJ805864	n.a.
Satyrinae	Melanitini	*Melanitis leda*	meled1	China: Yunnan, Hekou	KM111608	KJ777784	KJ805840	KJ777739	KJ805865	KJ777762
		*Melanitis phedima*	mephe1	China: Fujian, Dehua	KM111609	KJ777785	KJ805841	KJ777740	KJ805866	n.a.
	Elymniini	*Elymnias hypermnestra*	elhyp1	China: Yunnan, Hekou	KM111610	KJ777786	KJ805842	KJ777741	KJ805867	KJ777763
		*Elymnias malelas*	elmal1	China: Xizang, Motuo	KM111611	KJ777787	KJ805843	KJ777742	KJ805868	KJ777764
	Zetherini	*Callarge sagitta*	casag1	China: Gansu, Wenxian	KM111612	KJ777788	KJ805844	KJ777743	KJ805869	KJ777765
		*Ethope noirei*	NW121-7	Vietnam	DQ338773*	n.a.	n.a.	n.a.	DQ338915*	n.a.
		*Penthema adelma*	peade1	China: Gansu, Wenxian	EF534103*	KJ777789	KJ805845	KJ777744	KJ805870	n.a.
		*Penthema darlisa*	CP-B02	Vietnam	DQ338775*	n.a.	n.a.	n.a.	DQ338917*	n.a.
	Satyrini	*Lopinga achine*	loach1	China: Shaanxi, Baoji	KM111631	KJ777792	KJ805848	KJ777748	KJ805874	KJ777767
		*Hipparchia autonoe*	hiaut1	China: Qinghai, Huzhu	KM111644	KJ777794	KJ805850	KJ777750	KJ805876	KJ777769
		*Ninguta schrenkii*	nisch1	China: Shaanxi, Huoditang	KM111641	KJ777793	KJ805849	KJ777749	KJ805875	KJ777768
		*Lethe albolineata*	lealb1	China: Yunnan, Jinghong	KM111634	KJ777795	KJ805851	KJ777751	KJ805877	KJ777770
		*Tatinga tibetana*	tatib1	China: Shaanxi, Baoji	KM111633	KJ777796	KJ805852	KJ777752	KJ805878	KJ777771
		*Neope pulaha*	nepul1	China: Sichuan, Pingwu	KM111640	KJ777797	KJ805853	KJ777753	KJ805879	KJ777772
		*Mycalesis mamerta*	mymam1	China: Yunnan, Jinping	KM111627	KJ777798	KJ805854	KJ777754	KJ805880	KJ777773
		*Minois dryas*	midry1	China: Shaanxi, Baoji	KM111645	KJ777799	KJ805855	KJ777755	KJ805881	KJ777774
	Amathusiini	*Stichophthalma howqua*	sthow1	China: Yunnan, Hekou	AY218250*	KJ777790	n.a.	KJ777745	KJ805871	n.a.
		*Faunis aerope*	faaer1	China: Zhejiang, Danxi	n.a.	KJ777791	KJ805846	KJ777746	KJ805872	n.a.
		*Amathusia phidippus*	NW114-17	Indonesia	DQ018956*	n.a.	n.a.	n.a.	DQ018923*	n.a.
		*Thauria lathyi*	thlat1	China: Yunnan, Jinghong	KM111613	n.a.	KJ805847	KJ777747	KJ805873	KJ777766
		*Discophora necho*	NW101-6	Indonesia	DQ338747*	n.a.	n.a.	n.a.	DQ338887*	n.a.

Note: * indicates the sequence downloaded from GenBank; n.a. indicates the corresponding gene fragment is not available.

### DNA extraction, amplification and sequencing

Genomic DNA was extracted from 95–100% ethanol-preserved muscle tissue of two adult butterfly legs, using an EasyPure Genomic DNA Kit according to the manufacturer’s instructions (TransGen Biotech Co., Led., Beijing, China). Extracted genomic DNA was eventually dissolved in 80 µL ddH_2_O and kept in a freezer (–20 °C) until it was used for polymerase chain reaction (PCR). Sequences of six nuclear and mitochondrial genes (*EF-1α*, *28s rDNA*, *COI*, *COII*, *Cytb* and *16s rDNA*) were ampliﬁed through PCR in a total volume of 25 µL. The volume consisted of 12.5 µL CWBIO 2 × Taq MasterMix, 8.5 µL sterile distilled H_2_O, 2.0 µL genomic DNA template and 1.0 µL 10 µM each primer. The primers used and corresponding annealing temperature in PCR as well as references are listed in Table 2. After electrophoretic analysis to ensure the amplification products were the target fragments we needed, the PCR products were subsequently sent to Sunny Biotechnology Co., Ltd. (Shanghai, China) for sequencing with the same primers used in the PCR. All sequences gathered in this study have been deposited in the GenBank.

**Table 2.** Primers in PCRs for multiple genes used in this study.

Gene	Primer name (forward or reverse reading)	Sequence	Annealing temperature	References
*COI*	LCO1490 (f)	GGT CAA CAA ATC ATA AAG ATA TTG G	51 °C	Folmer et al. (1994)
	HCO2198 (r)	TAA ACT TCA GGG TGA CCA AAA AAT CA		Folmer et al. (1994)
*COII*	EVA (f)	GAG ACC ATT ACT TGC TTT CAG TCA CT	53 °C	Caterino and Sperling (1999)
	PATRICK (r)	CTA ATA TGG CAG ATT ATA TGT ATT GG		Caterino and Sperling (1999)
*Cytb*	CB-N3665 (f)	GTC CTA CCA TGA GGT CAA ATA TC	50 °C	Simon et al. (2006)
	CB-N11526 (r)	TTC AAC TGG TCG TGC TCC AAT TCA		Simon et al. (2006)
*16s rDNA*	LR-J-12887 (f)	CCG GTT TGA ACT CAG ATC ACG T	49 °C	Simon et al. (1994)
	LR-N-13398 (r)	CGC CTG TTT ATC AAA AAC AT		Simon et al. (1994)
*EF-1α*	ELF2F (f)	AAA ATG CCC TGG TTC AAG GGA	52 °C–57 °C	Wan et al. (2013)
	ef51.9 (f)	CAR GAC GTA TAC AAA ATC GG		Monteiro and Pierce (2001)
	efrcM4 (r)	ACA GCV ACK GTY TGY CTC ATR TC		Monteiro and Pierce (2001)
*28s rDNA*	rD3.2a (f)	AGT ACG TGA AAC CGT TCA SGG GT	58.8 °C	Whiting (2002)
	Rd4.2b (r)	CCT TGG TCC GTG TTT CAA GAC GG		Whiting (2002)

### Sequence analysis and phylogenetic inference

Sequence chromatogram was checked carefully using Chromas Pro software (Technelysium Pty Ltd., Tewntin, Australia). Each protein-coding sequence was translated for confirmation and assignment of codon positions in Primer Premier version 5.00 software (Premier Biosoft International, Palo Alto, CA). Multiple sequences were aligned using MAFFT version 7.037 with the auto strategy (Katoh and Standley 2013) and, if necessary, manual adjustment was made in MEGA version 6.06 (Tamura et al. 2013). Base frequency and the number of variable and parsimony informative sites were calculated in MEGA version 6.06 (Tamura et al. 2013). We investigated the chi-square of homogeneity of base frequencies across taxa for each gene with the program PAUP4.0b10 (Swofford 2002). The aligned ambiguous regions of two non-coding ribosomal genes (i.e. *16s rDNA* and *28s rDNA*) were retained because these positions might contain some information that is potentially useful for phylogenetic reconstruction (Aagesen 2004; Redelings and Suchard 2009). As proposed by Xia et al. (2003), we performed tests of substitutional saturation based on the *Iss* (i.e. index of substitutional saturation) statistic for different partitioned dataset with DAMBE version 5.3.74 (Xia 2013). For this method, if *Iss* is smaller than *Iss.c* (i.e. critical *Iss*), we can infer that the sequences have experienced little substitutional saturation (Xia and Lemey 2009).

Maximum likelihood (ML) analysis was performed using the raxmlGUI version 1.3 interface (Silvestro and Michalak 2012) of RAxML version 7.2.6 (Stamatakis 2006). The best-fit substitution model for each gene partition was determined by jModelTest version 2.1.4 (Darriba et al. 2012) under the Akaike Information Criterion (AIC) (Akaike 1974). Clade supports were assessed using the ML + rapid bootstrap algorithm with 1000 bootstrap iterations.

Bayesian inference (BI) analyses were conducted in MrBayes 3.1.2 (Ronquist and Huelsenbeck 2003). The best-fit partitioning schemes and partition-specific substitution models, defined from 16 subsets formed by gene and codon position of the six genes used, were tested using the ‘greed’ algorithm of program PartitionFinder v1.1 (Lanfear et al. 2012) under the Bayesian information criterion (BIC). Two independent MCMC runs were performed either for 300,000 generations or until the average standard deviation of split frequencies fell below 0.01. The sampling frequency was set as every 100 generations. After the first 25% of the yielded trees were discarded as burn-in, a 50% majority-rule consensus tree with the posterior probability (PP) values was constructed by summarizing the remaining trees. For BI analyses, two different datasets, the full six-gene-dataset and the non-*COI* + *Cytb* + *COII*-3rds-dataset (with 3rd positions removed), were used to examine the phylogenetic utility of the 3rd sites of *COI* + *Cytb* + *COII*, because these sites have suffered substantial saturation (see the results).

### Phylogenetic informativeness

We used phylogenetic informativeness (PI) proﬁles to quantify the relative contribution of each partition to the resulted tree. The peak of the PI distribution is suggested to predict the maximum phylogenetic informativeness for corresponding partition (Owen et al. 2014). The PI proﬁles were generated with the PhyDesign (Townsend 2007; Lopez-Giraldez and Townsend 2011). For this, the aligned sequences and an ultrametric tree are needed as input files. In the sequence file, the eight partition schemes identified by PartitionFinder v1.1 (Lanfear et al. 2012) were applied. The ultrametric tree was generated with the BEAST version 1.7.5 (Drummond et al. 2012) using the eight partitions and corresponding models determined by PartitionFinder v1.1 (Lanfear et al. 2012) as well.

## Results

### Sequence characterization

One hundred and fifty-four sequences of the six genes were obtained for 30 species (Tables 1, 3). The ﬁnal alignment yields 3,402 bp of the combined sequence data, of which 1,312 are variable and 1,053 are parsimony informative. The Chi-square test reveals no signiﬁcant base composition heterogeneity among the taxa for any gene fragment, even for the *28s rDNA* showing a high level of CG base composition (*p* = 0.138). In the case of the saturation test, all observed values of *Iss* are smaller than the *Iss.c* values for both symmetrical and asymmetrical topologies in all gene fragments. However, when the analysis was taken for each of the three codon positions of coding gene fragments separately, values of *Iss* for the third codons of all the *COI*, *COII* and *Cytb* genes are smaller than the *Iss.c* values in both symmetrical and asymmetrical topologies, indicating some of these sites have suffered substantial saturation.

**Table 3.** Sequence statistics for the six gene regions.

	*COI*	*COII*	*Cytb*	*16s rDNA*	*EF-1α*	*28s rDNA*
Number of sequences	29	25	25	26	30	19
Alignment length (bp)	621	690	591	530	510	460
Percentage A(%)	29.6	34.9	31.7	37.7	25.5	15.5
Percentage T(%)	39.5	41.6	43.3	41.6	26.1	18.2
Percentage C(%)	16.7	13.4	16.0	12.8	25.9	33.8
Percentage G(%)	14.2	10.1	9.0	7.9	22.5	32.5
Number of variable sites	233	288	275	167	165	184
Number of parsimony informative sites	203	222	226	125	139	138
Chi-square test of base frequency	*p* = 1.000	*p* = 1.000	*p* = 0.998	*p* = 1.000	*p* = 0.999	*p* = 0.138

### Model selection and phylogenetic reconstruction

Each gene partition shows the GTR + I + G for its best-fit substitution model except the *28s rDNA* being the GTR + G, but we imposed the GTR + G for all gene partitions in ML analysis as recommended by Zahiri et al. (2011). For BI analysis, the best partitioning scheme includes eight partitions. Each partition and corresponding parameters used in BI analyses are summarized in Table 4.

**Table 4.** The best-fit partitioning schemes and corresponding partition models used in BI analysis.

Partitioned dataset	Nucleotide model under BIC	Implemented parameters in BI analysis
1) *COI* 1st + *COII* 1st + *Cytb* 1st	GTR + I + G	nst = 6, rates = invgamma
2) *COI* 2nd + *COII* 2nd + *Cytb* 2nd	HKY + I + G	nst = 2, rates = invgamma
3) *COI* 3rd + *COII* 3rd + *Cytb* 3rd	HKY + G	nst = 2, rates = gamma
4) *16s rDNA*	GTR + I + G	nst = 6, rates = invgamma
5) *28s rDNA*	GTR + G	nst = 6, rates = gamma
6) *EF-1α* 1st	TrN + I	nst = 6, rates = inv
7) *EF-1α* 2nd	JC	nst = 1
8) *EF-1α* 3rd	GTR + G	nst = 6, rates = gamma

**Figure 1.**
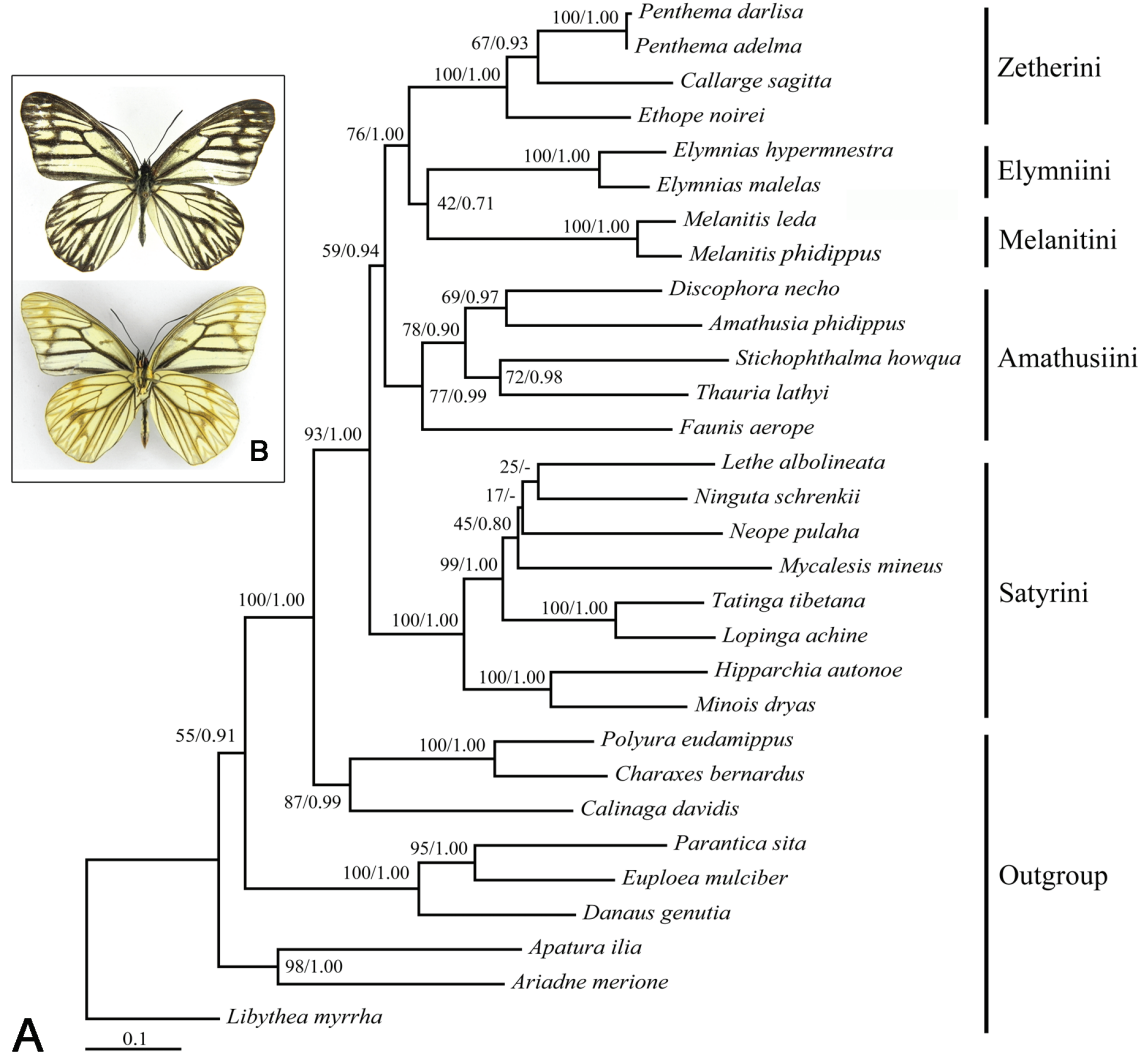
**A** Bipartitions tree obtained from maximum likelihood (ML) analysis based on the full six-gene-dataset; numbers separated by a slash on node are bootstrap value (BV) and posterior probability (PP) **B** *Callarge sagitta* (Leech), habitus, dorsal view on the above and ventral view on the below.

The ML and BI trees based on the full six-gene-dataset show generally identical topologies (summarized in Figure 1). All tribes included with two or more taxa examined in this study are recovered to be monophyletic mostly with strong support values. The traditional “satyrine” clade consisting of Calinaginae, Charaxinae and Satyrinae is well-recovered by strong bootstrap value (BV) 100 and PP 1.00. The five tribes of Chinese satyrines constitute the Satyrinae clade with BV 93 and PP 1.00. Within this clade, the Satyrini is consistently recovered as sister of others. Then, Amathusiini branches off, and the Zetherini is sister to the sister group (Melanitini + Elymniini), but the relationship between Melanitini and Elymniini is poorly supported by both ML and BI analyses (BV = 42, PP = 0.71). The genus *Callarge* is nested into the Zetherini, forming a sister group with *Penthema* Westwood.

**Figure 2.**
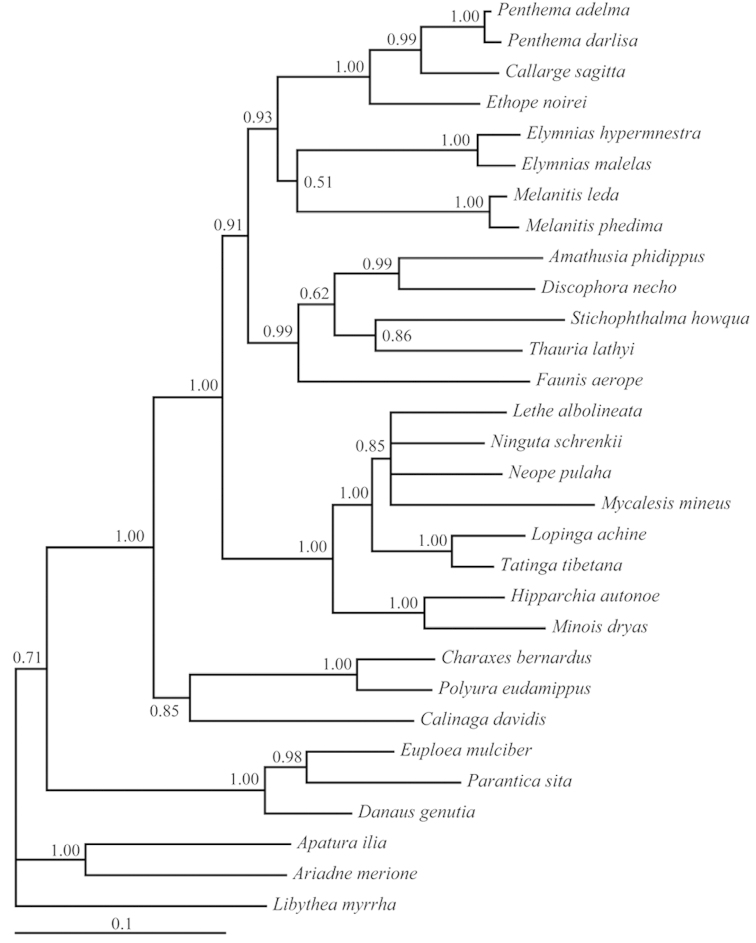
50% majority-rule trees obtained from Bayesian inference (BI) analyses based on the non-*COI* + *Cytb* +*COII*-3rds-dataset. Numbers on nodes are the posterior probabilities (PP).

The trees constructed based on the non-*COI* + *Cytb* + *COII*-3rds-dataset is shown in Figure 2. The tree shows generally same topology with that based on the full six-gene-dataset, but some nodes especially that describing the terminal taxa are less resolved. This indicates that the 3rd sites of *COI* + *Cytb* + *COII* provided poor supports for the tribe level relationships.

### Phylogenetic informativeness

**Figure 3.**
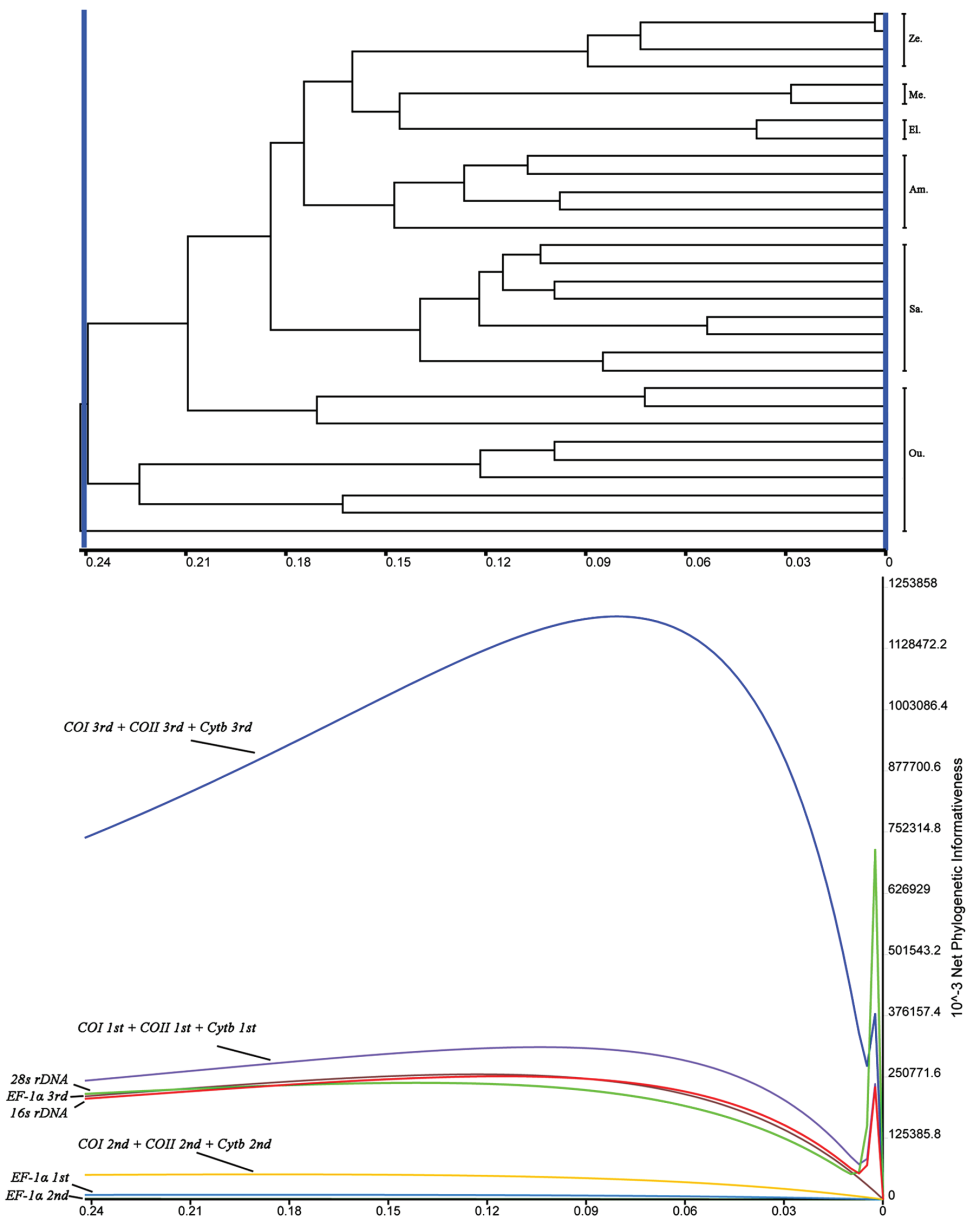
Phylogenetic informative proﬁles for all subsets used in this study. **Ze.** Zetherini; **El.** Elymniini; **Me.** Melanitini; **Am.** Amathusiini; **Sa.** Satyrini.

As shown in Figure 3, the 3rd codon positions of the combined *COI*, *Cytb* and *COII* has the highest phylogenetic signal at all taxonomic levels, and a peak of the PI distribution can be recognized at about the 1/3 position of the tree near the terminal branches. Followed are the 1st codon positions of the combined *COI*, *Cytb* and *COII*. The ribosomal *16s* and *28s* DNA generally show the same phylogenetic informativeness with the 3rd codon positions of *EF-1α*, especially on the zone of tree showing the tribe level relationships of the Satyrinae. The remaining 2nd codon positions of the combined *COI*, *Cytb* and *COII*, the 1st and 2nd codon positions of *EF-1α* show relatively limited phylogenetic signals.

## Discussion

### Phylogenetic informativeness of related genes

The studies of molecular systematics have been increasingly accessible because more genetic markers have been developed with the advances of sequencing technology. However, how to make informed choice to these markers confuses many systematics (Danforth et al. 2005). In high level systematics of Satyrinae, *EF-1α* was commonly used and proven to be quite informative in all previous studies (Peña et al. 2006; Peña and Wahlberg 2008; Wahlberg et al. 2009). Our results show the two ribosomal genes (i.e. *16s rDNA* and *28s rDNA*) have generally the same phylogenetic informativeness with *EF-1α* (Figure 3), which indicates that the former two genes also contribute well in constructing the tribe level relationships. Moreover, we infer the *28s rDNA* would show better informativeness if the *28s rDNA* sequence data for each sampling taxon had been obtained in this study. The consistency between the *28s rDNA* and *EF-1α* in phylogenetic utility supports the findings of Danforth et al. (2005) who suggested that the nuclear ribosomal and protein-coding genes should be combined in phylogenetic practices after comparing the substitution patterns between them in other groups of insects. The *16s rDNA* have been proven to be informative in high level systematics (e.g. Nazari et al. 2007) and was even recommended as standard marker for insect phylogenetics (Caterino et al. 2000). The high phylogenetic utility of *16s rDNA* examined in this study provides support for these proposals. However, this result does not support that mitochondrial gene datasets should not be applied on the deep divergences due to their substantial variation (Lin and Danforth 2004; Danforth et al. 2005).

We do not recommend the use of the 3rd positions of combined *COI*, *Cytb* and *COII* in high level systematics of Satyrinae, although these sites show higher phylogenetic signals than other partitions (Figure 3). On the one hand, our saturation tests show some sites of the 3rd positions of combined *COI*, *Cytb* and *COII* have suffered substantial saturation. These sites may positively contribute to the tip nodes of trees, but for the nodes after the PI profile peak they may become the source of noise deep in the tree and cause homoplasy (Owen et al. 2014). On the other hand, the deep branch pattern of BI tree (Figure 2) generally not change when excluding the 3rd positions of combined *COI*, *Cytb* and *COII*. This result indicates that the 3rd positions of combined *COI*, *Cytb* and *COII* contribute poorly to the tribe level relationships of the trees based on the full six-gene-dataset.

### Phylogenetic relationships among related tribes of Satyrinae

In this study, we present the first use of the ribosomal genes in reconstructing the tribe level relationships of the Satyrinae. The “satyrine” clade consisting of Calinaginae, Charaxinae and Satyrinae defined by Peña and Wahlberg (2008) and Wahlberg et al. (2009) are well-supported by our results. Moreover, monophyly of involved Satyrinae with the Amathusiini included is highly supported by all ML and BI analyses based on multiple outgroup taxa, which confirms, at least partially, the findings of Peña et al. (2006) who noted Satyrinae is monophyletic with inclusion of the tribes Morphini, Brassolini and Amathusiini of Morphinae (*sensu* Ackery et al. 1999) (Peña and Wahlberg 2008; Wahlberg et al. 2009).

Among the five tribes of Satyrinae analyzed, our results recover the Satyrini as the basal lineage with a long-branch split from the rest four tribes, in agreement with the findings of Peña et al. (2006) and Peña and Wahlberg (2008). However, relationships among the remaining four tribes are incongruent with other related studies regardless of the Dirini not included herein. Our results recover their relationships as Amathusiini + (Zetherini + (Elymniini + Melanitini)); whereas other related studies concluded the following relationships: (Elymniini + Melanitini) + (Zetherini + Amathusiini) in both ML and BI analyses of Wahlberg et al. (2009), the Elymniini + Melanitini + Zetherini + Amathusiini in MP analysis of Wahlberg et al. (2009), and the Melanitini + (Zetherini + (Elymniini + Amathusiini)) in BI analysis of Peña and Wahlberg (2008). Although ribosomal genes were used for the first time in our study, and both the ML and BI trees based on the full six-gene-dataset show identical topology, it should be noticed that the nodes in ML analysis describing the tribe level relationships are lowly- or moderately-supported. Therefore, the relationships among the related five tribes recovered herein need further verification based on more sampling taxa.

The monotypic genus *Callarge* is distributed restrictedly in China and on the northern border of Vietnam. Morphologically, this genus has marked black veins and lacks eyespots on wings. It is currently placed in Zetherini of Satyrinae (Chou 1999; Yuan et al. 2008) by the presence of hairless eyes, the wings without striking eyespots, and the forewing with basal part of vein Sc, posterior vein of discal cell and vein 2A not swollen (Chou 1998). For the first time, we verify the status of the genus based on molecular phylogenetic analyses, and reveal that it is sister to the *Penthema* in the present study.
